# A Neonatal Surprise: A Rare Round Ligament Cyst

**DOI:** 10.7759/cureus.77912

**Published:** 2025-01-24

**Authors:** Aqeela J Madan, Fayza Haider, Nabeel Al-Ashiri, Fatima Kadhem, Sayed Ali I Almahari

**Affiliations:** 1 Pediatric Surgery, Salmaniya Medical Complex, Manama, BHR; 2 Radiology, Salmaniya Medical Complex, Manama, BHR; 3 Pathology and Laboratory Medicine, Salmaniya Medical Complex, Manama, BHR

**Keywords:** congenital, mass, neonate, pediatric surgery, round ligament of liver

## Abstract

Ligamentum teres (or round ligament) of the liver is a cord-like structure found within the falciform ligament. Lesions originating from the ligaments of the liver are a rare entity, especially in the pediatric population; they can be either benign or malignant. Here we report a case of a neonatal abdominal mass arising from the round ligament of the liver that was treated with surgical excision, raising the question of possible outcomes if left untreated.

We present a case of a term female neonate born with an abdominal mass noticed after being investigated for feed intolerance; the mass was diagnosed with radiological imaging, surgically excised at the neonatal period, and sent for histopathology. It was found to be a benign cyst arising from the round ligament of the liver.

Here we discuss the radiological and pathological findings of such a rare mass that, to the best of our knowledge, has not been found to be reported in the pediatric literature. Symptoms can be variable and nonspecific; the need for investigation and type of imaging with surgical vs. conservative intervention all depend on the nature of the mass or cyst arising from the liver ligaments; the final histopathology can be benign or malignant; therefore, excision is important to reach a final diagnosis.

In conclusion, cysts of the round ligament of the liver in a neonate have yet to be described in the literature; the etiology and the possible future symptomatology, if left untreated, are still unknown.

## Introduction

Ligamentum teres (or round ligament) of the liver is found within the falciform ligament on the inner surface of the anterior abdominal wall and represents a remnant of the umbilical vein. It is a connecting venous structure between the placenta and the umbilical portion of the left portal vein located at the dorsal free margin of the falciform ligament [1].

Cysts of the round ligament of the liver are a rare entity, especially in the pediatric population; many differential diagnoses can arise, including benign or malignant pathologies such as lipomas, hydatid cysts, abscesses, or mesothelial cysts. Malignancy is rare but has been reported in the literature [2].

Here we report a case of a neonatal cyst arising from the round ligament of the liver. The radiological and pathological features of this cyst are also presented.

## Case presentation

This is a case of a female neonate that was born to a 43-year-old gravida 5 para 3 abortion 2 gestational diabetes mellitus (GDM), hypothyroid mother at 36 weeks gestation by normal vaginal delivery with a birth weight of 2.76 kg, she was found to have vomiting after her first feed, her vomitus was milk content only, non-bilious and non-projectile. The general examination was nonspecific as she was vitally stable, on room air, and not in distress, not pale, cyanosed, or jaundiced initially. Abdominal examination revealed a large abdominal mass that was mobile, moving upwards when lifting the umbilicus, smooth, firm, with no skin changes above it, the mass was then confirmed by imaging, no antenatal images were available for comparison as the mother was not consistent with follow up. Lab investigations including complete blood count (CBC), liver function test (LFT), renal function test (RFT), and tumor markers were all within normal limits.

**Figure 1:**
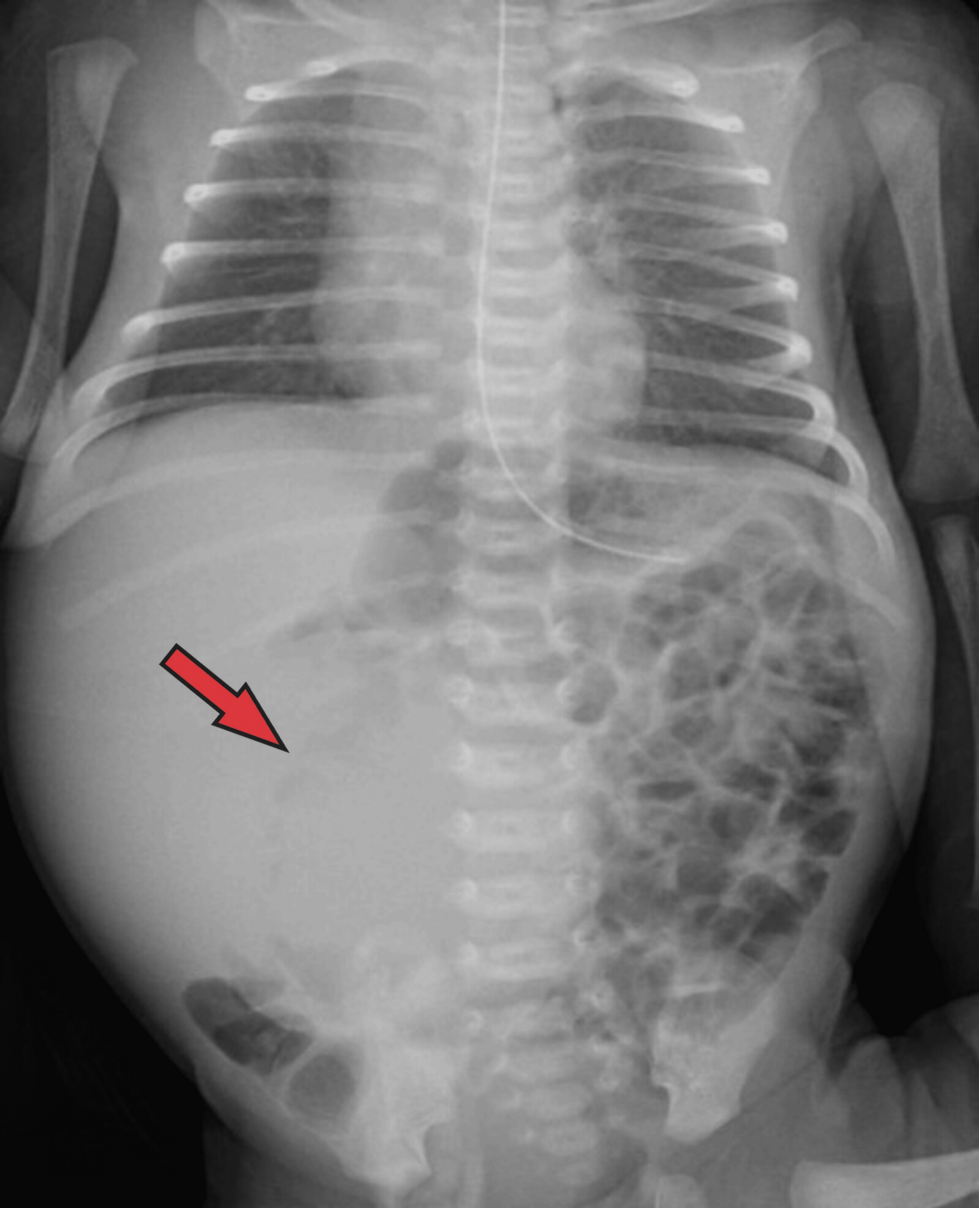
Asymmetry of the abdomen with large opacity was seen in the right hemiabdomen, deviating the bowel loops toward the left side. No calcification was noted. The thorax and bone were unremarkable.

A plain abdominal film (Figure 1) showed asymmetry of the abdomen with large opacity seen in the right hemiabdomen deviating the bowel loops toward the left side of the abdomen; no calcification was seen, and the thorax and bone were unremarkable.

**Figure 2:**
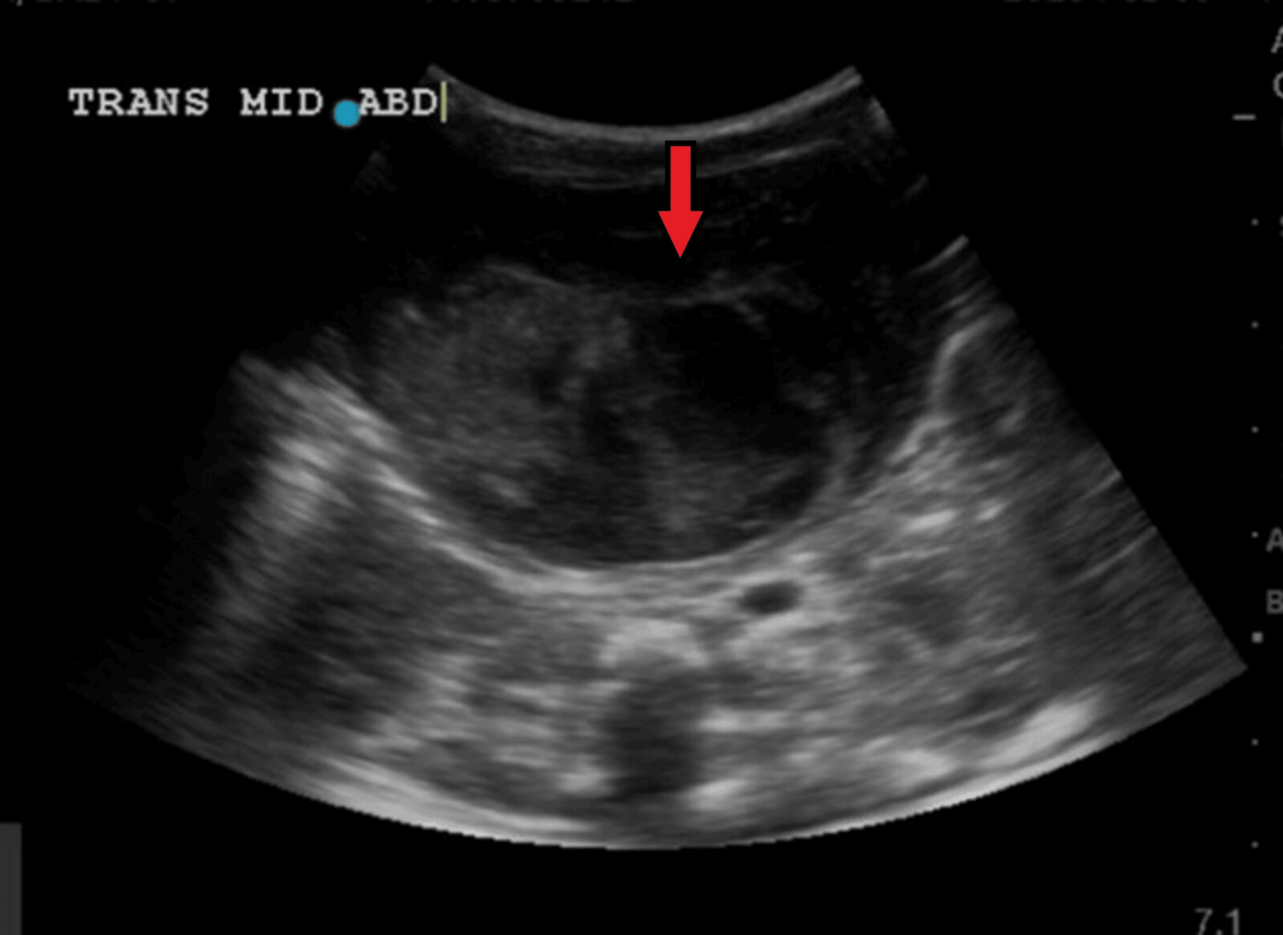
Abdominal ultrasound showed a large, complex cystic lesion occupying the mid-abdomen, more toward the right side.

An ultrasound of the abdomen (Figure 2) showed evidence of a large complex cystic lesion occupying the mid-abdomen more to the right side, the level of its origin could not be properly determined by the limited portable ultrasound done in the neonatal intensive care unit (NICU) and the radiologist recommended further evaluation by computer tomography (CT).

**Figure 3:**
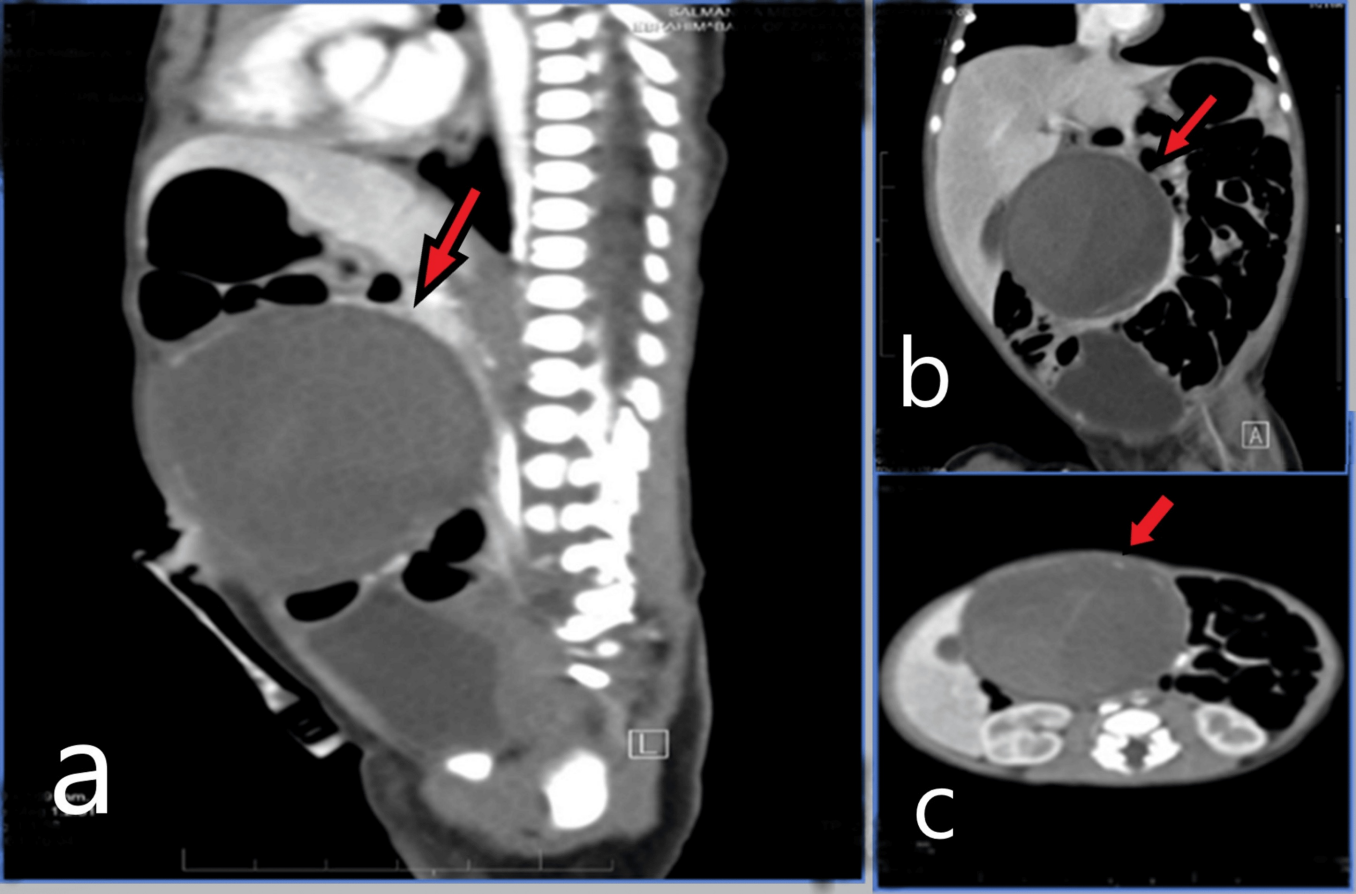
Cross-sectional CT images a. Sagittal image; b. Coronal image; c. Axial image The cuts demonstrated the complex cystic lesion, which was extending from the porta hepatis up the umbilicus anteriorly, which was exerting a mass effect on the adjacent structures. There was no invasion or encasement to the adjacent structures and the major abdomen organs were grossly normal.

A CT scan of the abdomen and pelvis showed a well-defined, large, complex cystic lesion at the mid-abdominal level (umbilical region) containing thick, enhanced septations, most likely representing a complicated/complex mesenteric cyst. The images (Figure 3) demonstrated a complex cystic lesion, which extended from the porta hepatis up the umbilicus anteriorly, exerting a mass effect on the adjacent structures. Although there was no invasion or encasement of the adjacent structures, the major abdominal organs were grossly normal. The other less likely differential diagnosis was pedunculated mesenchymal hamartoma from the inferior surface of the liver.

The need for surgical intervention was explained to the parents due to the following reasons: the mass was large (greater than 5 cm in diameter), it was causing pressure symptoms, the baby was not tolerating feeds, and it was required to arrive at a final diagnosis to define the origin of the mass.

The patient was taken for operative intervention on post-natal day nine.

**Figure 4:**
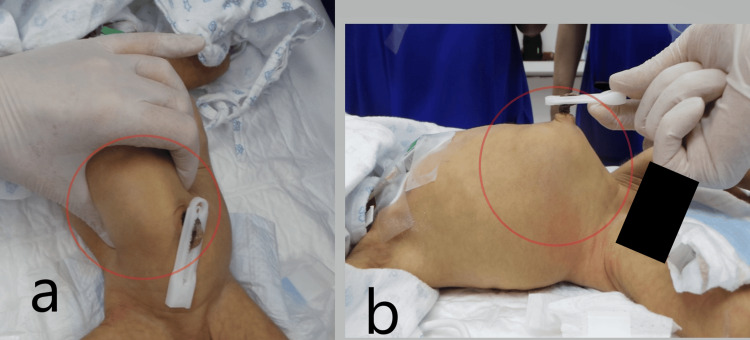
Intraoperative images a. Examination of the abdominal mass under general anesthesia; b. The mass was noticed to be elevating while lifting the umbilicus.

Upon examination under anesthesia (Figure 4a), there was evidence of jaundiced skin and mucus membranes, a visible and palpable right abdominal mass raised upwards while pulling the umbilical cord (Figure 4b), mobile and smooth, semisolid, up to the liver.

**Figure 5:**
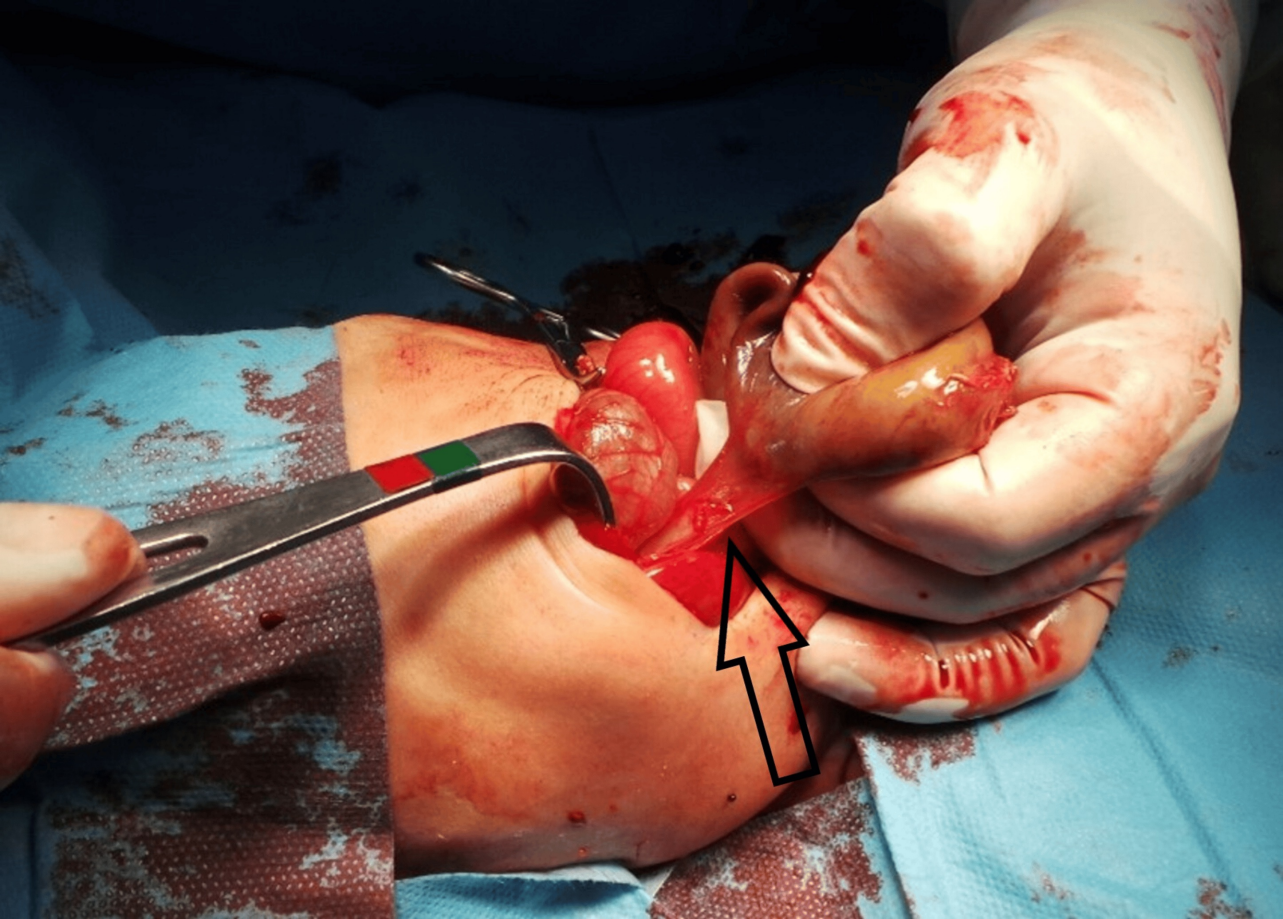
Dissection of the cyst from the surrounding structures revealed that it originated from the liver's round ligament.

**Figure 6:**
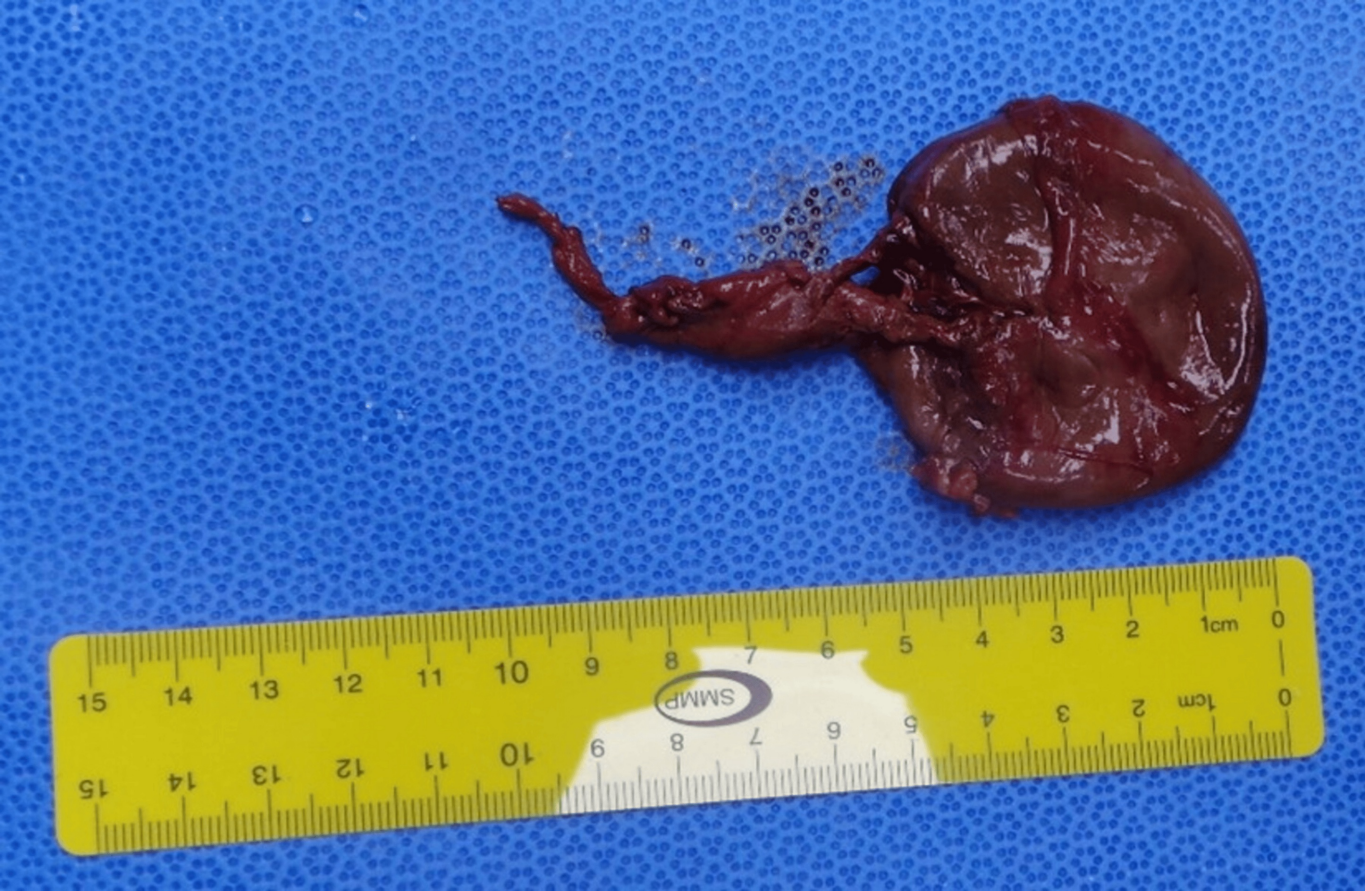
Final specimen of the cyst after the evacuation of the content measuring around 5 cm

During the exploratory laparotomy, the mass was found to be adherent to the peritoneum and had a capsule around it. A cyst dissected from the surrounding structures, clearly originating from the round ligament of the liver, was tense and ruptured during dissection, releasing blood clots and old blood (Figure 5). The mass was a large 5*5 cm cyst containing clotted old blood, with no evidence of solid structures seen, weighing 24 grams (Figure 6).

The liver, gallbladder, small and large bowel, and urinary bladder were all unremarkable.

The patient’s recovery was uneventful; she was discharged from the NICU on the fifth postoperative day. She tolerated full feeds, and the wound completely healed. Postoperative tumor markers were negative.

**Figure 7:**
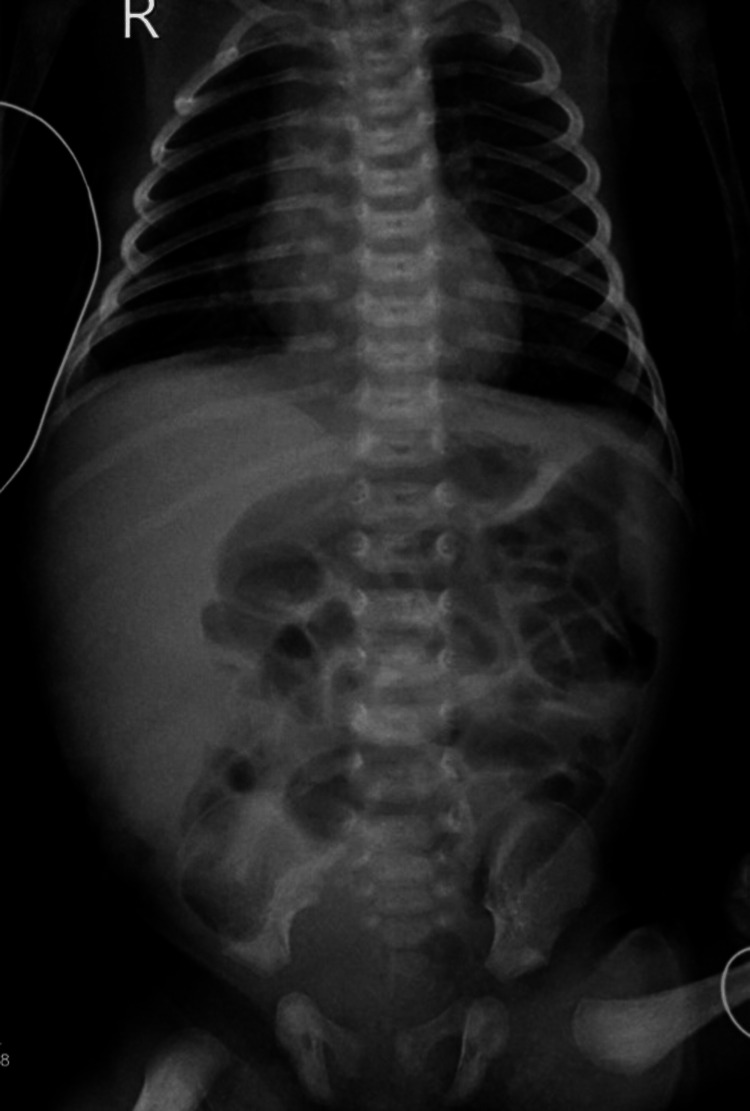
A postoperative X-ray of the abdomen showed a normal position of the bowel loops with normal opacity of the abdominal organs.

**Figure 8:**
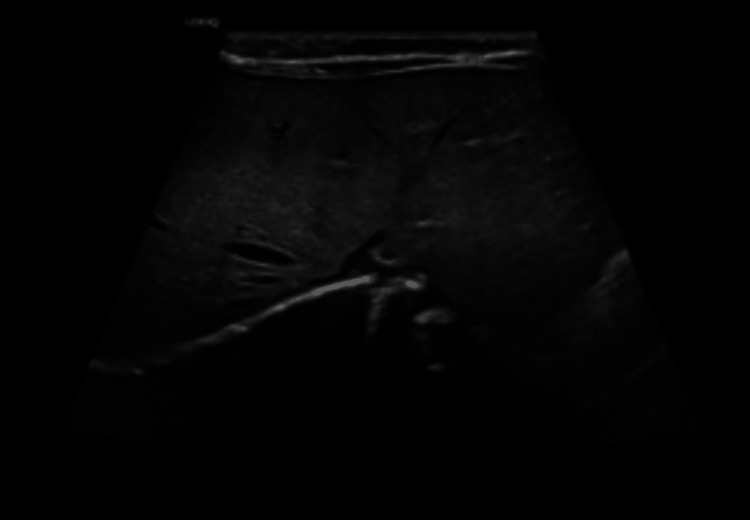
Postoperative ultrasound of the abdomen at the porta hepatis shows a normal liver with complete resection of the lesion.

A postoperative abdominal plain film showed the normal position of the bowel loops with normal opacity of the abdominal organs (Figure 7). An ultrasound of the abdomen repeated after three weeks reported no evidence of local recurrence or any other abdominal or pelvic pathology. An image of the porta hepatis showed a normal liver with complete resection of the lesion (Figure 8).

**Figure 9:**
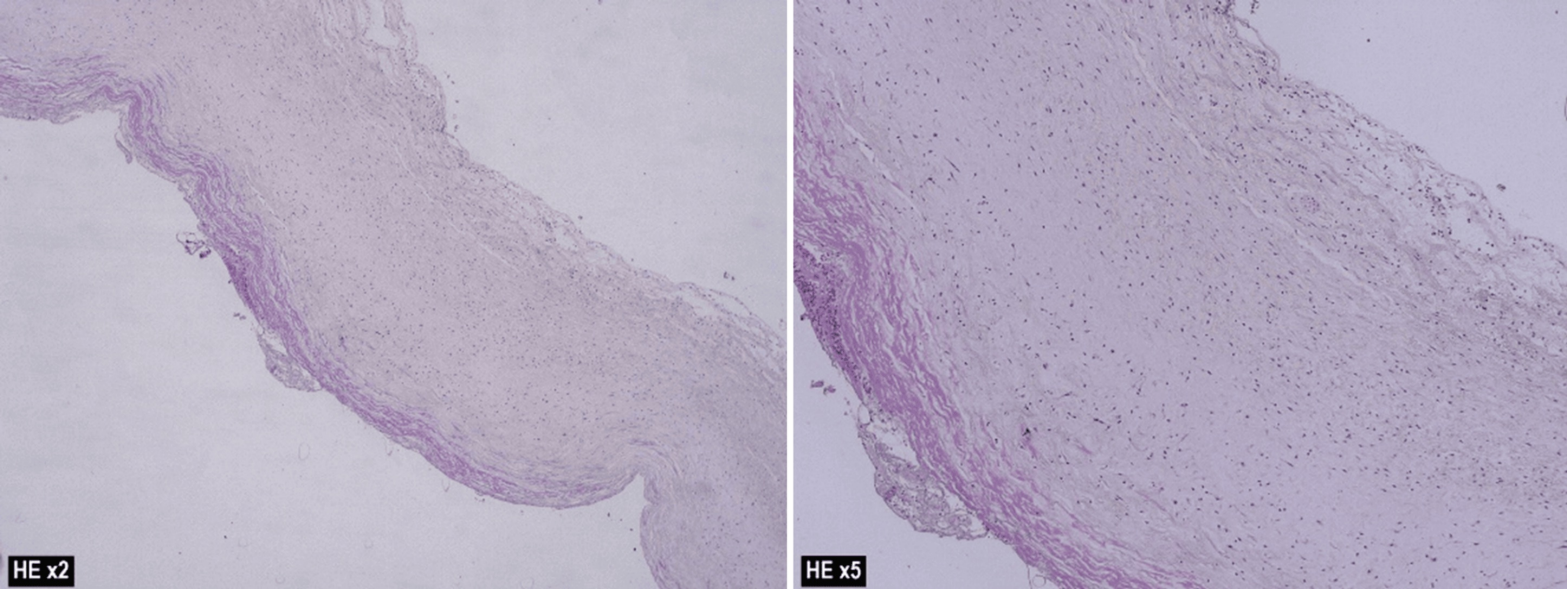
A dilated vascular channel lined by attenuated intima covered by fibrin was noted. The media revealed focal calcifications with a thinned-out smooth muscle layer (hematoxylin and eosin stain, 20x and 50x magnification).

**Figure 10:**
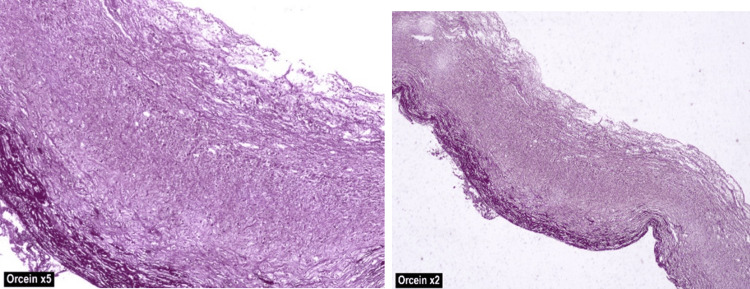
Attenuated internal/external elastic fibers confirmed the venous nature of the vascular channel (orcein stain, 20x and 50x magnification)

The final histopathological diagnosis was a ligamentum teres cyst (Figure 9), which showed evidence of a dilated vascular channel lined by attenuated intima covered by fibrin. The media reveals focal calcifications with a thinned-out smooth muscle layer. Figure 10 shows the attenuated internal/external elastic fibers, which confirmed the venous nature of the vascular channel. The features were compatible with cystic dilatation of the umbilical vessel.

## Discussion

Round ligament cysts are a rare entity and have been scarcely reported in the literature [1,3], with only a few cases reported in adults and no reported cases in the neonatal period [2]. These cysts can be benign or possibly malignant. 

Ligament cysts can present with pressure symptoms if the size is large enough to cause intestinal symptoms like partial or complete obstruction with vomiting or obstipation. Other symptoms like vomiting, pain, and fullness were reported in adults [1,3].

Imaging is a great source of help when it comes to diagnosing abdominal mass in general, as it can define the origin of the mass as well as the content, cystic or solid, simple or complex, and the exact size and location. Starting with ultrasound is always advocated in pediatrics as it has less radiation and can help define the location and nature of the mass [4]; if more details are needed, then CT or MRI is our next approach accordingly [5]. An MRI needs general anesthesia and is not preferably used unless required in pelvic or soft tissue and skeletal masses. Hydatid cysts must be considered in the differential diagnosis of abdominal masses even though they are nearly non-existent in newborns [6].

Indications for surgical intervention depend on the size of the mass and the signs and symptoms that the patient presents with. Early intervention is required if the patient is symptomatic or if the lesion is suspected to be malignant from the imaging results [7]. An approach of follow-up and observation, mainly in cysts of ovarian origin, is considered by some if the mass is less than 5 cm in size and is asymptomatic with a simple cyst as the most likely diagnosis and the benign features on imaging support the conservative approach. As liver ligament cysts are rarely found in the pediatric population, it is wise to raise the question of whether observation will lead to symptoms later in the future or if many missed lesions of the falciform ligament are not reported due to their asymptomatic nature. An open approach is the most appropriate approach in neonates; however, a laparoscopic approach can be considered in older children and adults [4]. The laparoscopic approach appears safe, feasible, and less invasive without compromising surgical principles and today should be considered the gold standard in most cases [8].

It is indeed important to completely excise any suspicious lesion to reach a final diagnosis and avoid any complications in the future or risks of missing malignancy.

## Conclusions

Cysts of the round ligament of the liver in neonates are rare and have yet to be described in the literature; they are easily diagnosed with modern-day radiological investigations. Moreover, surgical resection is the mainstay therapeutic approach, as histopathological analysis is the main final diagnostic tool of any congenital abdominal mass; the etiology and possible future symptomatology if left untreated are still unknown. 
